# Crosstalk between Immune Cells and Adipocytes Requires Both Paracrine Factors and Cell Contact to Modify Cytokine Secretion

**DOI:** 10.1371/journal.pone.0077306

**Published:** 2013-10-21

**Authors:** Carolina Franco Nitta, Robert A. Orlando

**Affiliations:** Department of Biochemistry and Molecular Biology, University of New Mexico, School of Medicine, Albuquerque, New Mexico, United States of America; Universidade Federal do Rio de Janeiro, Brazil

## Abstract

Increased adiposity results in a heightened infiltration of immune cells into fat depots, which in turn generates a pro-inflammatory phenotype in obese individuals. To better understand the causal factors that establish this pro-inflammatory profile, we examined events leading to crosstalk between adipocytes and immune cells. Using isolated spleen-derived immune cells, stimulated with LPS, together with cultured adipocytes, we differentiated the effects of paracrine factors and cell-cell contact on TNFα, IL-6 and MCP-1 secretion levels and secretion profiles. When splenocytes and adipocytes were co-cultured without direct contact, permitting only paracrine communication, secretion of IL-6 and MCP-1 were increased by 3- and 2.5-fold, respectively, over what was secreted by individual cultures, whereas TNFα secretion was reduced by 55%. When cells were co-cultured with direct cell-cell contact, IL-6 and MCP-1 secretion were increased by an additional 36% and 38%, respectively, over that measured from just paracrine stimulation alone, indicating that cell contact provides a synergistic signal that amplifies elevated cytokine secretion stimulated by paracrine signals. Using splenocytes from TNFα^-/-^ mice showed that the absence of TNFα has little effect on paracrine stimulation of cytokine secretion, but attenuates cell contact-mediated enhancement of IL-6 and MCP-1 secretion. Furthermore, TNFα supports cell contact-mediated signaling in part, but not exclusively, through Nuclear Factor-κB activation. These findings indicate that engagement of cell contact between immune cells and adipocytes, in conjunction with locally secreted paracrine factors, activates a unique signaling pathway that mediates crosstalk between these cell types leading to marked effects on cytokine secretion and profile.

## Introduction

Obesity has reached epidemic proportions as a universal health challenge and is now firmly established as a substantial risk factor for developing atherosclerotic and hypertensive cardiovascular diseases, as well as type II diabetes mellitus [1]. Lean adipose tissue contains several cell types that together contribute to normal adipose tissue function, including endothelial cells that supply proper oxygenation and nutrient delivery, fibroblasts that contribute to interstitial matrix deposition, and resident macrophages that provide an immunologic surveillance function. Curiously, with the onset of obesity the cell type profile within growing adipose tissue changes during excessive weight gain largely due to a substantial infiltration of inflammatory macrophages [2], [3] and, as recently discovered, other immune cells such as T and B cells [4]–[10]. The unique or combined roles of these immune cell types in obese adipose tissue is not yet known. No evidence has been presented pointing to tissue infection that would provide homing signals for circulating immune cells, although suggestions have been put forward that tissue injury due to anoxia and apoptosis or necrosis within rapidly expanding adipose tissue may trigger macrophage recruitment [11]–[14]. The fact that inflammatory macrophages can account for up to 40% of the total cell population within obese adipose tissue, affirms that this a substantial physiological response [2].

Current thought maintains that the primary trigger for macrophage recruitment into obese adipose tissue is mainly due to heightened secretion of MCP-1 (monocyte chemoattractant protein-1) [15]–[18], which is followed by secretion of other cytokines, such as tumor necrosis factor-alpha (TNFα), interleukin-6 (IL-6) and interleukin-1β (IL-1β). As a result, these secreted factors establish a low-level, chronic, systemic inflammation among obese individuals [2], [3]. This chronic inflammatory profile is thought to alter normal signal transduction events [19], and in doing so, establish a mechanistic link between several multi-faceted metabolic diseases, such as hyperlipidemia, hypertension, obesity-dependent cardiovascular diseases and type II diabetes mellitus [20]–[23], by altering normal signal transduction events.

To better understand the contributions of chronic inflammation in obesity to these metabolic diseases, it is vital to define the cytokine expression profile of immune cells and adipocytes within inflamed adipose tissue and identify how paracrine and autocrine activities influence this profile. Some reports have suggested that cytokine production is limited to infiltrating macrophages, yet other studies have offered a more complex picture that involves intercellular communication between macrophages and adipocytes. For example, murine (3T3-L1 cells) or human (SGBS) adipocytes incubated with macrophage-conditioned media increases mRNA expression and protein levels of inflammation-related genes, including MCP-1 and IL-6 [24]–[26]. Reverse stimulation also occurs in which macrophages cultured with adipocyte-conditioned media increase their expression of IL-6 and TNFα [26]. These findings suggest that both cell types contribute to elevated cytokine expression by co-stimulating in a paracrine manner with secreted factors found in their respective culture media. We have recently confirmed and extended this paracrine communication activity by showing that cultured adipocytes can independently respond to TNFα stimulation by increasing IL-1β, IL-6 and COX-2 (cyclooxygenase) expression through nuclear factor-κB (NF-κB) signaling [27]. Alternative to conditioned media, co-culture methods have been used to explore macrophage-adipocyte intercellular communications. For this approach, collagenase-treatment was used to disaggregate cells in excised adipose tissue followed by differential centrifugation to separate buoyant adipocytes from more dense stromal vascular cells. This stromal vascular fraction contains a variety of cell types, including endothelial cells, fibroblasts, pre-adipocytes and macrophages; however, in an obese setting, it also contains a highly enriched inflammatory macrophage population [2], [3]. Unfortunately, the standard methodology for adipose tissue cell separation has led to mixed, sometimes conflicting results; one study reported that the infiltrating macrophage population is responsible for almost all TNFα expression[2], with IL-6 being expressed by both populations [2], [28], while other groups concluded that IL-6 was released mainly by non-fat cells [29], [30]. A third group reported that the stromal vascular fraction showed greater MCP-1 expression than the adipocyte fraction, concluding that macrophages are the main source of this chemokine in adipose tissue [31], [32]. One likely explanation of these conflicting observations comes from a parallel study indicating that collagenase treatment, combined with the isolation procedure used for fractionating adipose tissue into macrophage and adipocyte cell populations can significantly alter cytokine expression profile by artificially inducing inflammatory mediators and down-regulating adipocyte-specific genes [33].

In the present study, we have investigated the contributions of paracrine activities and cell-cell contact between immune cells and adipocytes on pro-inflammatory cytokine production. To circumvent the problems associated with tissue disaggregation, we have developed a trans-well co-culture model using primary murine splenocytes and 3T3-L1 cell-derived mature adipocytes. Use of splenocytes provides a better representation of the immune cell population found in obese adipose tissue [34], [35] and ensures normal intracellular signaling patterns, rather than introducing possible complications from altered signal transduction events likely present in immortalized transformed monocytic cell lines such as THP-1 or RAW294.7. Our findings provide novel evidence that when immune cells and adipocytes are cultured together, both diffusible, paracrine factors and cell-cell contact contribute synergistically in modifying TNFα, IL-6 and MCP-1 secretion. We also provide evidence identifying which cytokine is expressed by each cell type and demonstrate a functional role for TNFα in providing signaling that potentiates the cell contact effects on cytokine secretion.

## Materials and Methods

### Animals and Animal Care

Male C57Bl/6J (Stock #000664), GFP (C57Bl/6-Tg(UBC-GFP)30Scha/J; ubiquitous expression of Green Fluorescent Protein; Stock #004353) and TNFα ^-/-^ (B6.129S-Tnf^tm1Gkl^/J; Stock #005540) mice were purchased from Jackson Laboratories at 8 weeks of age. Animals were housed 2 per cage in a pathogen-free environment on a 12 h light/dark cycle and were provided free access to food and water. Mice were euthanized by CO_2_ asphyxiation and processed immediately for spleen removal. All procedures in this study were approved by the Institutional Animal Care and Use Committee of the University of New Mexico.

### Splenocyte isolation

Splenocyte isolation was performed according to Kruisbeek [36] using wild type C57Bl/6J, GFP-expressing C57Bl/6J, or TNFα ^-/-^ mice. After spleens were excised, they were placed in Dulbecco's Modified Eagle Medium (DMEM) supplemented with 10% Fetal Bovine Serum (FBS), 1 mM sodium pyruvate, 2 mM L-glutamine, 100 µg/mL streptomycin sulfate and 100 units/mL penicillin (complete DMEM). Spleens were homogenized into single cell suspensions by gently disaggregating tissue between frosted ends of two microscopy slides, filtered through a 100 µm cell strainer, and then centrifuged at 800× g for 3 min at 4°C. Supernatants were discarded and cell pellets were resuspended in 1 mL ACK (Ammonium-Chloride-Potassium) Lysis Buffer (150 mM NH_4_Cl, 10 mM KHCO_3_ and 0.1 mM Na_2_-EDTA; pH 7.4) for 5-10 min to remove contaminating red blood cells. Complete DMEM was then added and cells were centrifuged again at 800× g for 3 min at 4°C. Supernatants were removed; cells were resuspended in complete DMEM and used for co-culture experiments after cell densities were determined by hemocytometer counting.

### Cell Culture and Adipocyte Differentiation

3T3-L1 pre-adipocytes were purchased from American Type Culture Collection (ATCC, Manassas, VA, USA) and were cultured and differentiated according to Gonzales and Orlando, 2008 [27]. Briefly, cells were grown in complete DMEM media at 37°C with 5% CO_2_ and passaged twice weekly. For differentiation, cells were seeded into 6-well cell culture plates coated with 1% gelatin. When cells reached confluency, they were incubated with 250 nM dexamethasone, 450 µM 3-Isobutyl-1-methylxanthine and 167 nM insulin for 3 days, then thoroughly rinsed with culture media and incubated for an additional 3–4 days with 167 nM insulin. Differentiation was confirmed by morphological changes, including intracellular lipid droplet accumulation, as confirmed by microscopic observation.

### Adipocyte and Splenocyte Co-cultures

3T3-L1 cells were grown to confluency in 6-well culture plates and differentiated to mature adipocytes as described above. Adipocytes were then co-cultured with isolated splenocytes from wild type C57Bl/6J, GFP-expressing C57Bl/6J or TNFα ^-/-^ mice in direct and indirect contact systems in the presence or absence of LPS (*E. coli* 0111:B4 – 1 µg/mL; Sigma Aldrich, St. Louis, MO) in complete DMEM. For controls, cells were cultured individually with or without LPS, or cultured together in the absence of LPS. For indirect co-cultures, cells were cultured in a transwell system, with differentiated 3T3-L1 cells in the lower chamber and splenocytes (1.5×10^6^ cells) seeded in a 0.4 µm hanging cell insert (Millipore, Billerica, MA). The hanging insert is constructed with a membrane having pores that are large enough to permit the passage of small molecules, yet small enough to prevent the passage of even the most motile of cell types. For direct co-cultures, splenocytes (1.5×10^6^ cells) were added to differentiated 3T3-L1 cells, allowing direct contact between the two cell types. For TNFα recovery studies, co-cultures were supplemented with 0, 0.3 or 10 ng/mL purified murine TNFα (Cell Signaling, Danvers, MA). In NF-κB inhibitor experiments co-cultures were incubated with 2 µM Bay11-7082 (Calbiochem, La Jolla, CA).

### Cytokine ELISAs

Murine TNFα, IL-6, IL-1β, MCP-1 and IL-10 levels in individual cultures and co-culture supernatants were measured by ELISA Ready-Set-Go kit (eBioscience, San Diego, CA) according to the manufacturer's directions. Media samples were diluted accordingly to ensure samples were within the detection kit sensitivity, specifically for MCP-1 and IL-6.

### Fluorescence-activated cell sorting

After 24 h of direct co-culturing of 3T3-L1 cells and splenocytes from GFP mice, cells were sorted by fluorescence-activated cell sorting (FACS). Co-cultures were trypsinized and centrifuged at 800× g for 3 min at 4°C. Supernatants were removed and cells were resuspended in complete low serum (0.5% FBS) DMEM media with 5 mM EDTA and passed through a 100 µm cell strainer (BD Falcon) to ensure single-cell suspensions. GFP-positive and negative cells were sorted using the Beckman Coulter Legacy MoFlo high-speed sorter into separate tubes for subsequent mRNA extraction.

### RNA Isolation and Quantitative RT-PCR Analysis

Sorted 3T3-L1 cells and splenocytes were homogenized using the QIAshredder (Qiagen, Valencia, CA) and total RNA was isolated with RNeasy Mini Kit (Qiagen, Valencia, CA) according to manufacturer's recommendations. RNA was converted into cDNA using the High Capacity cDNA Reverse Transcription Kit (Applied Biosystems). Levels of F4/80, IL-6, adiponectin, MCP-1, TNFα and IL-10 were measured by quantitative Real-Time PCR (qRT-PCR), which was carried out using the LightCycler 480 SYBR Green I Master Mix chemistry (Roche Diagnostics, Indianapolis, IN) and analyzed on the LightCycler 480 instrument (Roche Diagnostics, Indianapolis, IN). Information on primer sequences, annealing temperatures, fragment sizes and genes can be found in Table 1. The reaction cycling parameters for IL-10 were performed as a 3-step qRT-PCR with a pre-incubation step at 95°C for 5 min and amplification for 45 cycles at 95° for 10 sec, 60°C for 15 sec and 72°C for 1 sec. All other genes were amplified in a 2-step qRT-PCR reaction with pre-incubation at 95°C for 15 min, followed by 40 cycles of amplification at 95°C for 15 sec and the annealing temperature (Table 1) for 1 min. A melting curve analysis was performed in each experiment for all genes to confirm specificity of single-target amplification. Gene expression changes were calculated using the relative standard curve method [37] and 36B4 mRNA levels were used as a normalizer. All samples were amplified in triplicate.

**Table 1:** Gene and primer information for qRT-PCR.

Gene	Primer sequences	Annealing temperature	Fragment size	GenBank no.
F4/80	Forward – GCTGTGAGATTGTGGAAGCA	66°C	136 bp	NM_010130
	Reverse – CTGTACCCACATGGCTGATG			
IL-6	Forward – AGTTGCCTTCTTGGGACTGA	60°C	191 bp	NM_031168
	Reverse – CAGAATTGCCATTGCACAAC			
Adiponectin	Forward – GGAACTTGTGCAGGTTGGAT	63°C	293 bp	NM_009605
	Reverse – CGAATGGGTACATTGGGAAC			
MCP-1	Forward – TCACCTGCTGCTACTCATTCACCA	60°C	98 bp	NM_011333
	Reverse – TACAGCTTCTTTGGGACACCTGCT			
TNFα	Forward – ACGGCATGGATCTCAAAGAC	63°C	116 bp	NM_013693
	Reverse – GTGGGTGAGGAGCACGTAGT			
IL-10	Forward – ATGCAGGACTTTAAGGGTTACTTG	60°C	254 bp	NM_010548
	Reverse - TAGACACCTTGGTCTTGGAGCTTA			
36B4	Forward – AAGCGCGTCCTGGCATTGTCT	60–66°C	136 bp	NM_007475
	Reverse – CCGCAGGGGCAGCAGTGGT			

36B4 annealing temperature varied according to target gene being analyzed. IL-6, interleukin-6; IL-10, interleukin-10; MCP-1, monocyte chemoattractant protein-1; TNFα, tumor necrosis factor alpha; and 36B4, 60S acidic ribosomal protein P0.

## Results

### Direct contact between splenocytes and adipocytes alters secreted inflammatory cytokine levels

**Figure 1:**
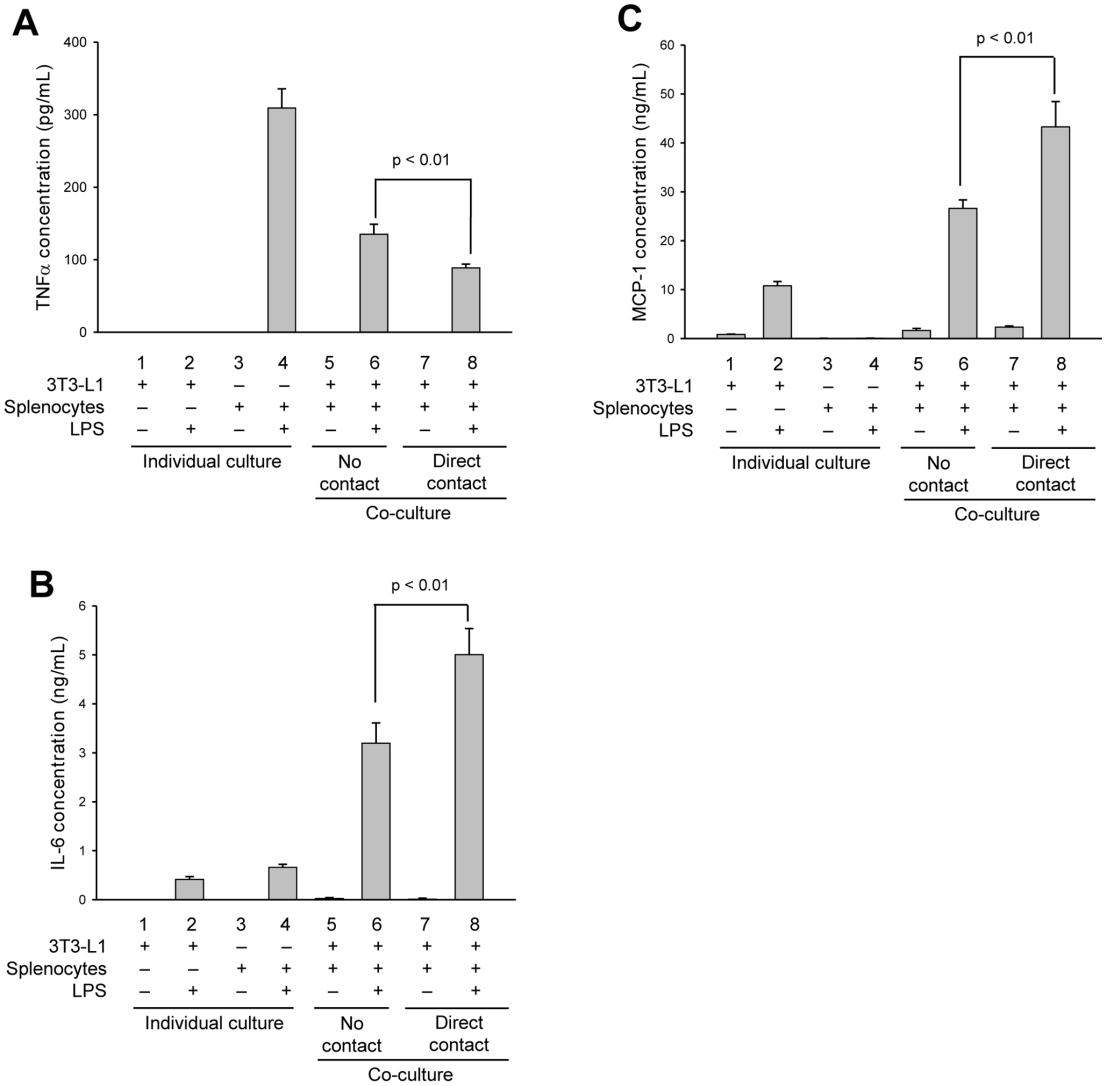
Direct contact between splenocytes and adipocytes alters secreted levels of inflammatory cytokines. Differentiated 3T3-L1 adipocytes (columns 1 and 2) or isolated murine splenocytes (columns 3 and 4) were cultured alone or together with either direct contact (columns 7 and 8) or no contact (cells separated by a 0.4 µm transwell filter system; columns 5 and 6). Cells were additionally incubated in the absence (-) (columns 1, 3, 5 and 7) or presence (+) (columns 2, 4, 6 and 8) of LPS (1 µg/mL) for 24 h. Secreted cytokines, TNFα (A), IL-6 (B) and MCP-1 (C), were quantified by capture ELISA. All experimental points were measured in triplicate for calculation of means and standard deviations. Comparison between individual cultures, co-cultures with no contact and co-cultures with direct cell-cell contact were calculated using ANOVA and followed by the post-hoc Bonferroni test. A significant effect was accepted when p<0.05.

To first determine if cytokine secretion is regulated by paracrine effects (no cell contact) or if cell-cell contact between adipocytes and immune cells contributes to the inflammatory response, we performed co-culture studies using 3T3-L1-derived adipocytes and isolated murine splenocytes, and assessed the quantity and pattern of TNFα, IL-6 and MCP-1 secretions. Cells were co-cultured either in transwells to mimic indirect contact, permitting only paracrine communications through diffusible factors, or cultured together to allow for direct cell-cell contact communications along with paracrine effects. Control studies included adipocytes and splenocytes incubated alone with or without LPS, to measure the effect of LPS stimulation on adipokine or cytokine secretion in each cell type without influence of co-culture, and adipocyte-splenocyte co-cultures without stimulation by LPS, to determine if non-activated cells can affect adipokine and cytokine secretion. Significant differences in TNFα, IL-6 and MCP-1 secretions were found when comparing indirect and direct adipocyte-splenocyte co-cultures indicating that cell-cell contact does indeed contribute to adipokine and cytokine secretions (Fig. 1). For TNFα, co-culturing appears to dampen secretion. In individual cultures, LPS stimulates TNFα secretion in splenocytes (Fig. 1A), but has no effect on adipocytes (Fig. 1A, compare columns 1 and 2). Co-culturing cells in transwells, to allow only paracrine communication, reduced TNFα secretion by 55% from that measured in LPS-stimulated splenocytes alone (Fig. 1A, compare columns 4 and 6). When cells were co-cultured with direct contact, TNFα secretion was reduced by an additional 33% as compared to paracrine effects (Fig. 1A, compare columns 6 and 8; p<0.01) demonstrating that paracrine factors and cell-cell contact both contribute to attenuate TNFα secretion. Also noteworthy, when adipocytes and splenocytes were co-cultured in the absence of LPS activation, whether indirect or direct culture, no measureable TNFα secretion was found (Fig. 1A, columns 5 and 7), suggesting that activation of inflammatory immune cells is required to mediate the effects seen in obese adipose tissue.

For IL-6, co-culturing of adipocytes and LPS-activated splenocytes appears to enhance secretion (Fig. 1B). When each cell type was cultured individually in the presence of LPS, some IL-6 secretion was measured for both adipocytes (Fig. 1B, compare columns 1 and 2) and splenocytes (Fig. 1B, compare columns 3 and 4). In contrast to the effects seen on TNFα secretion, when cells were co-cultured in transwells to measure the effects of paracrine stimulation, IL-6 secretion was increased by ∼3-fold over that measured for the summation of individual LPS-activated cultures (Fig. 1B, compare columns 2+4 with column 6). When cells were co-cultured with direct contact, IL-6 secretion was increased by an additional 36% over that measured for paracrine effects alone (Fig. 1B, compare columns 6 and 8; p<0.01). Similar to the effects seen on TNFα secretion, very little or no IL-6 secretion could be measured in the absence of LPS activation (Fig. 1B, columns 1, 3, 5 and 7).

In a similar manner as IL-6, co-culturing of adipocytes and LPS-activated splenocytes appears to have a substantial effect in enhancing MCP-1 secretion (Fig. 1C). In individual cultures, no measureable MCP-1 secretion could be detected in splenocytes with or without LPS stimulation (Fig. 1C, see columns 3 and 4). LPS treatment of adipocytes alone was able to induce some MCP-1 secretion (Fig. 1C, compare columns 1 and 2) and this secretion level increased by 2.5-fold when co-cultured with splenocytes indirectly in transwells (Fig. 1C, compare columns 2 and 6). When adipocytes and splenocytes were co-cultured with direct contact, MCP-1 secretion increased by an additional 38% over that measured for just paracrine stimulation (Fig. 1C, compare columns 6 and 8; p<0.01).

As an additional note, there were no detectable levels of IL-1β in our co-culture system under any of the conditions tested (data not shown).

### Paracrine stimulation and direct contact differentially effect cytokine secretion in a time-dependent manner

**Figure 2:**
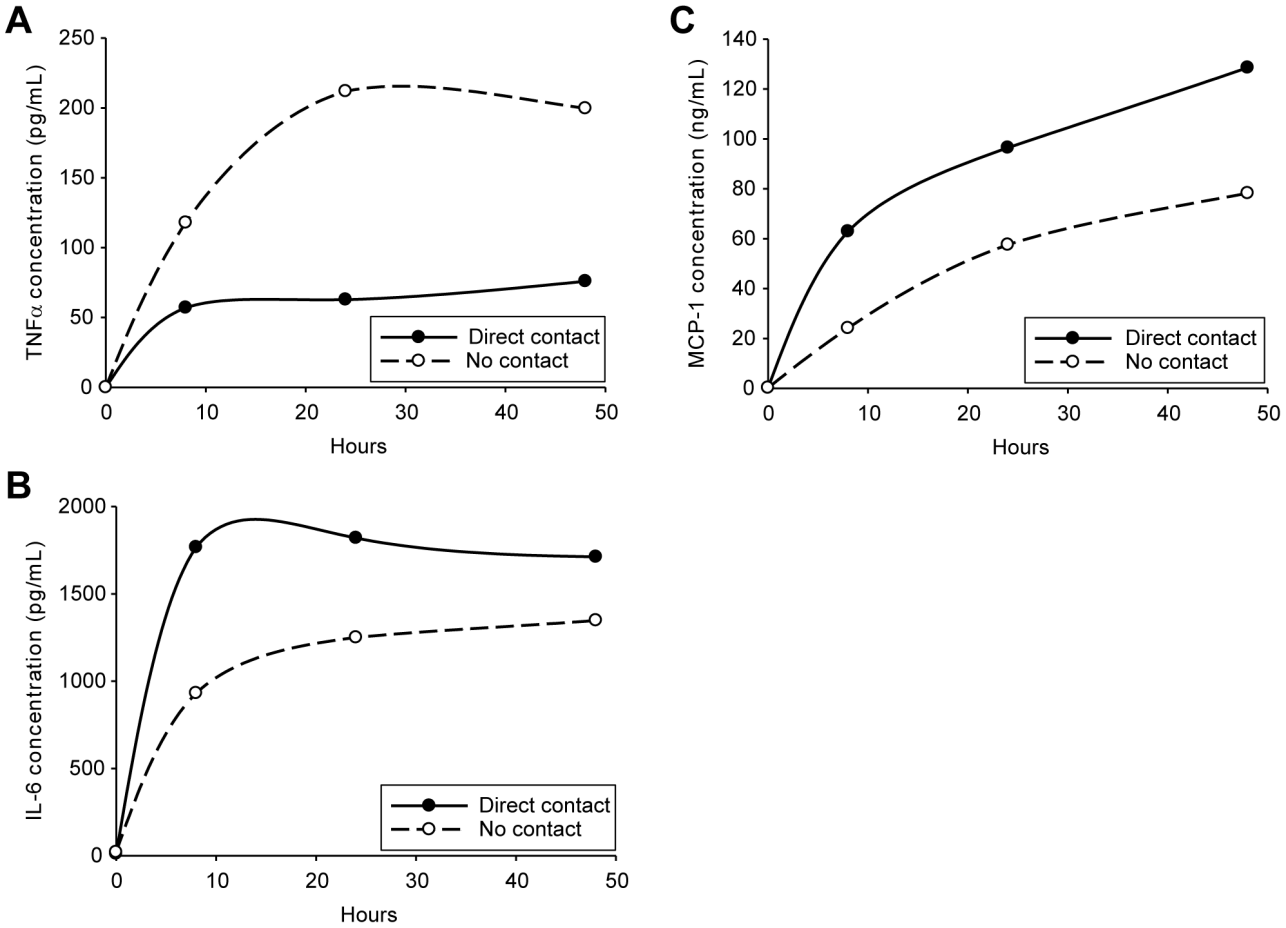
Rates of cytokine secretion and overall levels are affected by direct contact between adipocytes and splenocytes. Differentiated 3T3-L1 adipocytes and isolated murine splenocytes were co-cultured with no contact (separated by a 0.4 µm transwell filter system) (dashed lines) or with direct contact (solid lines) and incubated with LPS (1 µg/mL) for 0, 8, 24 and 48 h. Cytokines, TNFα (A), IL-6 (B) and MCP-1 (C), were measured in culture media following these incubation times by capture ELISA. All experimental points were performed in triplicate.

To better define the changes in cytokine secretion patterns resulting from paracrine factors alone or these factors together with direct cell-cell contact, we chose to perform a time course study. Co-culturing of activated splenocytes and adipocytes without direct cell-cell contact led to a time-dependent increase in secreted TNFα levels, reaching a maximal effect at approximately 20 h (Fig. 2A). When cells were cultured in direct contact, TNFα secretion was substantially reduced from levels measured from paracrine stimulation alone, reaching maximal secretion in <8 h.

Paracrine factors stimulated both IL-6 and MCP-1 secretion in co-cultures in a time-dependent manner, with maximal secretion of IL-6 recorded at approximately 20 h (Fig. 2B) and >48 h for MCP-1 (Fig. 2C). With direct cell-cell contact, stimulation of IL-6 secretion was greater than that measured for paracrine factors alone with a maximal effect at <8 h. The effect of cell-cell contact on increasing MCP-1 secretion was also rapid; however, the stimulatory effect did not reach a maximum even after 48 h of culture. These data demonstrate that paracrine factors, resulting from splenocyte-adipocyte co-culture, affect cytokine secretion in a time-dependent manner. Moreover, when cells were cultured under conditions allowing for direct cell-cell contact, the effects on cytokine secretion were enhanced over what was measured for paracrine factors alone.

### Pro-inflammatory cytokine expression profile for co-cultured splenocytes and adipocytes

**Figure 3:**
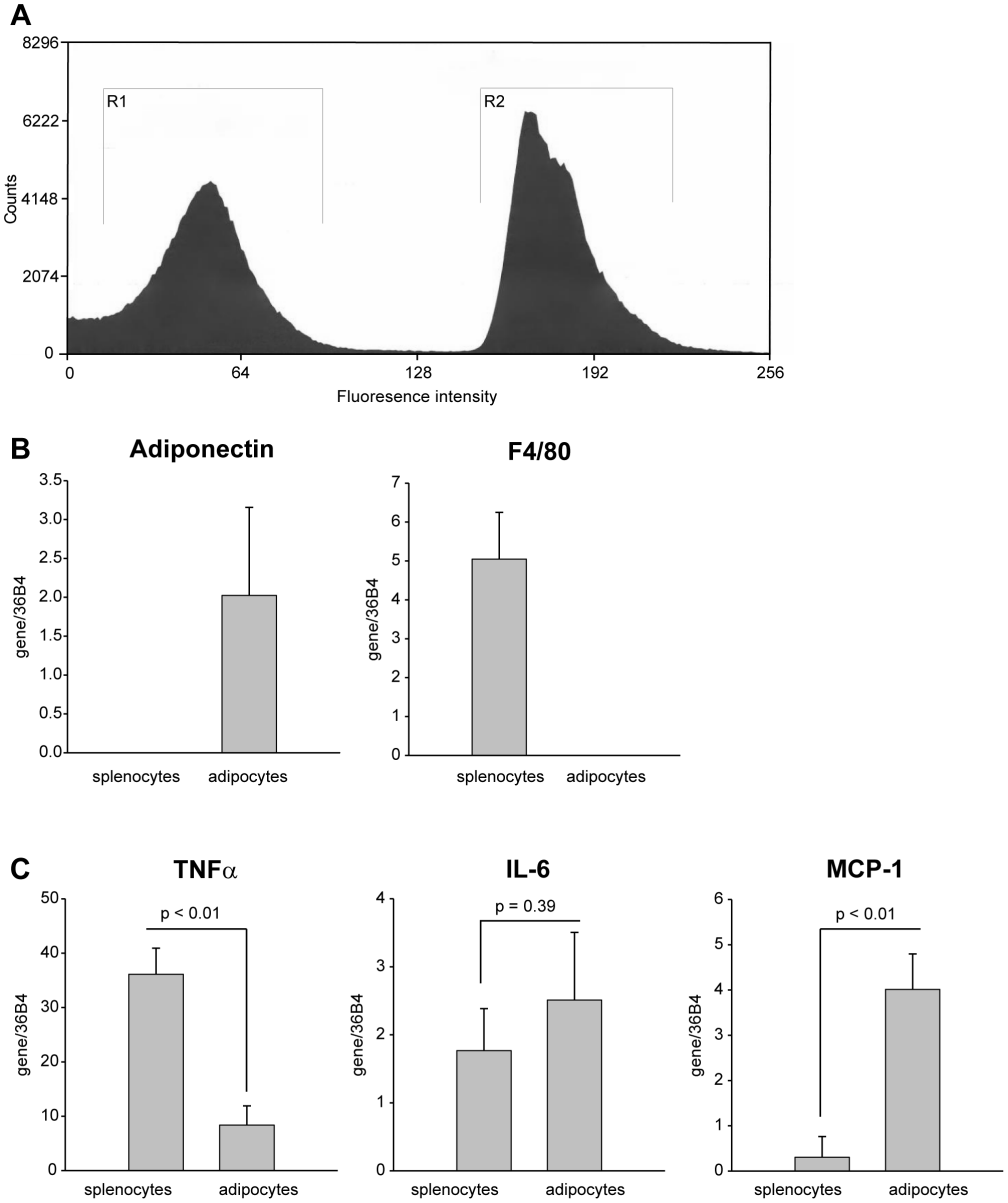
Splenocytes and adipocytes differentially express pro-inflammatory markers. Differentiated 3T3-L1 adipocytes were co-cultured in direct contact with GFP-expressing murine splenocytes and activated by incubation with LPS (1 µg/mL) for 24 h. Cells were sorted for GFP-positive (splenocyte) and negative (adipocyte) cells by FACS. (A) Representative FACS is shown, with GFP-negative cells gated in R1 and GFP-positive cells gated in R2. (B) Quantitative real-time PCR (qRT-PCR) was used to measure adiponectin and F4/80 expression, specific markers for adipocytes and splenocytes, respectively, to confirm efficiency of cell sorting. (C) TNFα, IL-6 and MCP-1 mRNA expression levels were quantified by qRT-PCR in splenocytes and adipocytes following cell sorting to distinguish individual cytokine expression patterns. All qRT-PCR values were normalized to values obtained for 36B4, a ribosomal 60S subunit protein. Experimental points were measured in duplicate (B and C) for calculation of means and standard deviations. In (C), statistical significance of differing secretion levels between splenocytes and adipocytes was determined by an unpaired two-tailed Student's t-test. A significant effect was accepted when p<0.05.

The next objective was to determine which cell type was responsible for expression of TNFα, IL-6 and MCP-1 following co-culturing. We chose to limit this evaluation to assessing mRNA expression levels because discriminating protein expression would require further individual culturing of the cells after co-culturing conditions and this extended incubation is known to artificially affect cytokine expression [33], [38]. For this analysis, cells were co-cultured with direct cell-cell contact and then separated by fluorescence-activated cell sorting (FACS), followed by quantification of mRNA expression for TNFα, IL-6 and MCP-1 in each cell type. In order to separate splenocytes from adipocytes using FACS, we performed the co-culturing incubation with splenocytes isolated from mice constitutively expressing green fluorescent protein (GFP) in all cell types. The data shown in Figure 3A is representative of a FACS profile for adipocytes and GFP-splenocytes (gates R1 and R2, respectively). With this approach, we are able to clearly separate GFP-expressing splenocytes from non-fluorescent 3T3-L1 adipocytes following co-culturing. To confirm the relative purity of each cell population with a more sensitive assay, we examined mRNA expression for cell specific markers in each cell type: F4/80 for macrophages (splenocytes) and adiponectin for adipocytes. As seen in Figure 3B, F4/80 expression was found only in the GFP-splenocyte population and adiponectin expression was found only in the adipocyte cell population.

Messenger RNA quantification for each cytokine revealed that, following their co-culture with direct cell-cell contact, adipocytes expressed relatively small quantities of TNFα mRNA, whereas splenocytes generated 4.5-fold greater levels than adipocytes (Fig. 3C). The opposite expression pattern was found for MCP-1; its expression was almost undetectable in splenocytes, whereas substantial expression was measured in adipocytes (Fig. 3C). Importantly, both adipocytes and splenocytes contribute equally to IL-6 expression following co-culture conditions (Fig. 3C), which is in contrast to previous studies which have suggested that the stromal vascular cells (largely macrophages) are the sole contributors of cytokines in obese adipose tissue [29], [31], [38], [39].

### TNFα signaling is necessary for cell contact-mediated increases in IL-6 and MCP-1 secretion

**Figure 4:**
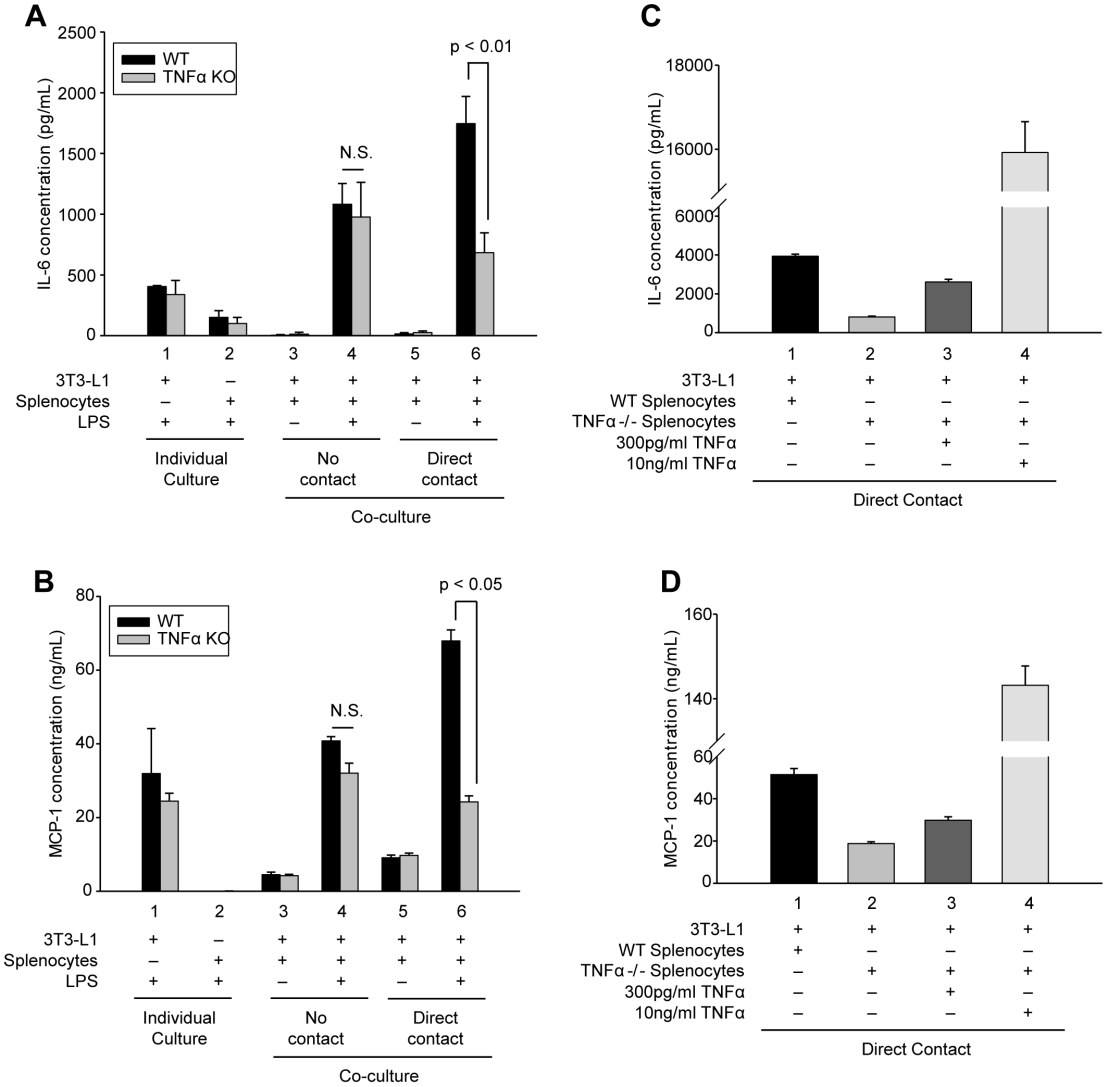
Cell contact-mediated enhancement of IL-6 and MCP-1 secretion requires TNFα signaling. (A and B) Differentiated adipocytes or murine splenocytes (black bars, isolated from wild type mice; gray bars, isolated from TNFα ^-/-^ mice) were cultured alone (individual culture, columns 1 and 2) or in co-culture with no contact (columns 3 and 4) or direct contact (columns 5 and 6) as in Figure 1. Cells were incubated in the absence (−) or presence (+) of LPS (1 µg/mL) for 24 h as indicated. (C and D) Wild type (WT) or TNFα ^-/-^ (TNFα KO) splenocytes were incubated with adipocytes with direct contact in the presence of LPS (1 µg/mL) and co-cultures were supplemented with 0, 300 pg/mL or 10 ng/mL purified murine TNFα as indicated. Secreted IL-6 (A and C) and MCP-1 (B and D) were quantified by capture ELISA. Experimental points were measured in triplicate (A and B) or duplicate (C and D) for calculation of means and standard deviations. For (A and B) comparison between individual cultures, co-cultures with no contact and co-cultures with direct cell-cell contact from WT and TNFα KO were calculated using ANOVA and followed by the post-hoc Bonferroni test. For C and D all experimental points were compared statistically by ANOVA followed by the Bonferroni post-hoc test, and differed significantly (p<0.01). N.S. denotes no statistical difference.

The data presented above establish that crosstalk between splenocytes and adipocytes, in the form of paracrine factors and cell-cell contact, significantly influences cytokine secretion in our in vitro model of inflamed adipose tissue. Of these cytokines, TNFα is known to be one of the major paracrine factors expressed in obese adipose tissue [40], [41]. Based on this observation, we examined if the paracrine activity of TNFα influenced the cell-cell contact mediated changes to IL-6 and MCP-1 secretion measured in our splenocyte-adipocyte co-cultures. To determine this, we repeated the co-culture study shown in Figure 1 with splenocytes derived from TNFα ^-/-^ mice. As expected, TNFα ^-/-^ splenocytes failed to express TNFα in the absence or presence of LPS (data not shown); however, with LPS-activation they did express IL-6 at levels similar to wild type splenocytes (Fig. 4A, column 2) confirming their functional response to LPS stimulation.

When TNFα ^-/-^ splenocytes were co-cultured with adipocytes in the absence of LPS activation, either with direct or no contact, little or no measureable IL-6 (Fig. 4A, compare columns 3 and 5) or MCP-1 (Fig. 4B, compare columns 3 and 5) could be detected (grey bars), similar to what was seen when using wild type splenocytes (black bars). With LPS activation, co-cultures of TNFα ^-/-^ splenocytes and adipocytes without direct contact demonstrated similar increases in IL-6 secretion as measured for wild type splenocytes (Fig. 4A, column 4, compare black and grey bars), indicating that TNFα activity is not required for paracrine-mediated enhancement of IL-6 secretion. For MCP-1, co-culture of LPS-activated TNFα ^-/-^ splenocytes and adipocytes without direct contact resulted in enhanced MCP-1 secretion when compared with individual adipocyte cultures (Fig. 4B, compare columns 1 and 4); however, similar to data obtained for IL-6, no statistical difference was measured in co-cultures of adipocytes with wild type splenocytes or TNFα ^-/-^ splenocytes (Fig. 4B, column 4, compare black and grey bars).

A very different effect on IL-6 and MCP-1 secretion was found when LPS-activated TNFα ^-/-^ splenocytes were co-cultured with adipocytes with direct cell-cell contact. The additional enhancement of IL-6 and MCP-1 secretion due to direct cell contact between TNFα ^+/+^ splenocytes and adipocytes is attenuated in co-cultures of TNFα ^-/-^ splenocytes and adipocytes (Fig. 4A and B, column 6, compare black and grey bars). The addition of exogenous TNFα to co-cultures of TNFα ^-/-^ splenocytes and adipocytes restored the contact-mediated enhancement in a dose-dependent manner for both IL-6 (Fig. 4C) and MCP-1 (Fig. 4D). These findings indicate that, although TNFα contributes little to the paracrine-mediated enhancement of IL-6 and MCP-1 secretion, its activity is necessary for cell contact-mediated augmentation of IL-6 and MCP-1 secretion.

### Secretion of anti-inflammatory factor, IL-10, is unaltered by paracrine factors or direct cell contact

**Figure 5:**
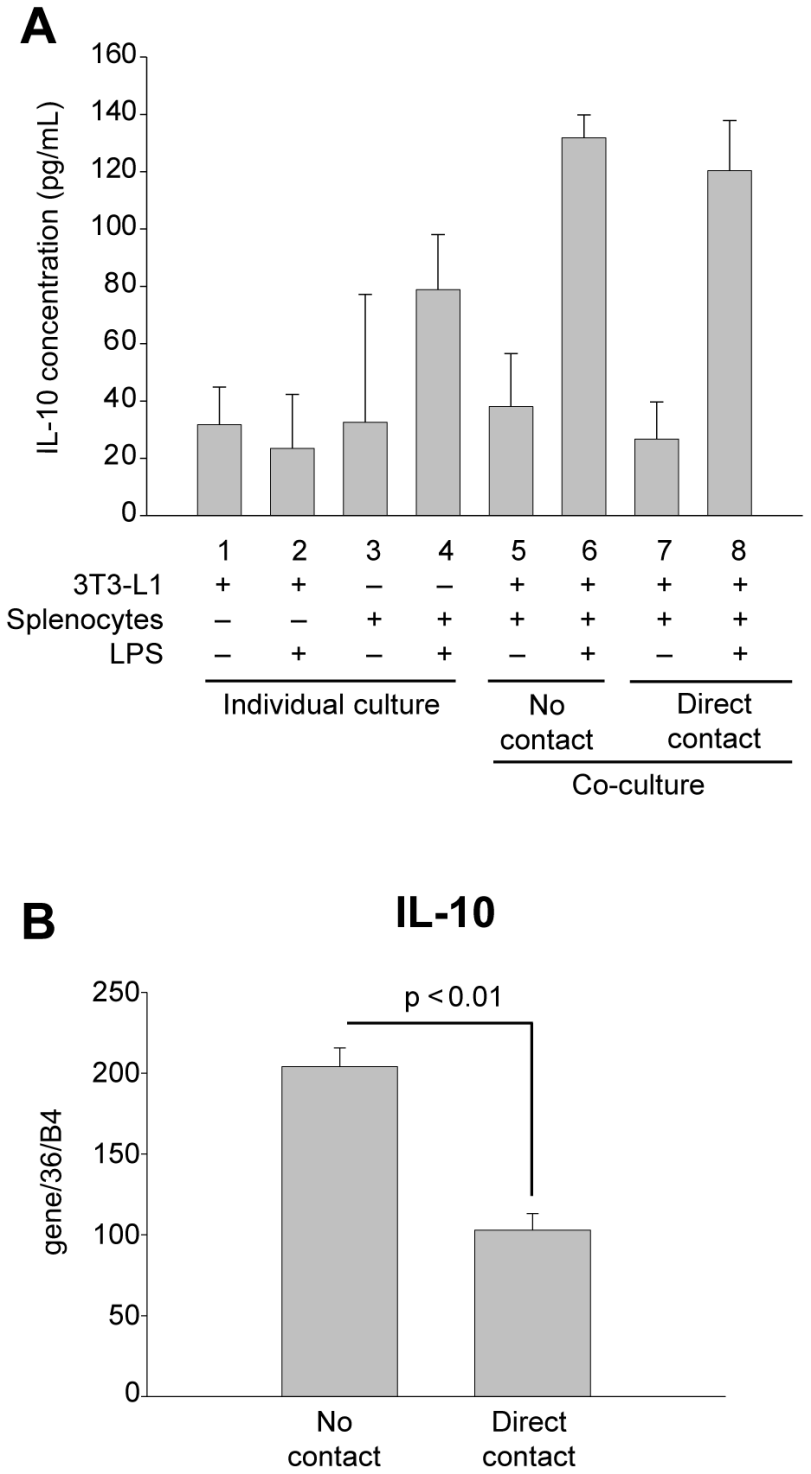
Effects of paracrine factors and cell contact on IL-10 secretion and expression. (A) Differentiated 3T3-L1 adipocytes or wild type murine splenocytes were cultured alone (columns 1 and 2 or 3 and 4, respectively) or together with either no contact (columns 5 and 6) or direct contact (columns 7 and 8). Cells were incubated in the absence (-) (columns 1, 3, 5 and 7) or presence (+) (columns 2, 4, 6 and 8) of LPS (1 µg/mL) for 24 h as indicated. Interleukin-10 (IL-10) in culture media was quantified by capture ELISA. (B) Differentiated 3T3-L1 adipocytes were co-cultured with no contact or direct contact with GFP-expressing murine splenocytes as in Figure 3 and activated by incubation with LPS (1 µg/mL) for 24 h. Splenocytes were sorted as GFP-positive cells by FACS and IL-10 mRNA expression was measured by qRT-PCR. qRT-PCR values were normalized to values obtained for 36B4. In (A) experimental points were measured in triplicate for calculation of means and standard deviations. Comparison between all conditions was calculated using ANOVA followed by the post-hoc Bonferroni test. No statistical significance was found between any measured points (p>0.05). For (B), experimental points were performed in duplicate, and a statistical comparison between no contact and direct contact was made using an unpaired two-tailed Student's t-test.

Inflamed adipose tissue is also known to express the anti-inflammatory cytokine, IL-10. Its secretion is thought to be in response to the elevated state of inflammation within obese adipose tissue [42], [43]. Because of this, the anti-inflammatory properties of IL-10 may provide a counterbalancing effect to dampen the actions of inflammatory cytokines [44]. Consequently, we examined the effects of paracrine factors and direct contact on IL-10 secretion in our co-culture system. In individual cultures, a small but measureable amount of IL-10 could be detected in non-stimulated and LPS-stimulated adipocytes (Fig. 5A, columns 1 and 2), while a 2.5-fold greater amount was found in cultures of LPS-stimulated splenocytes (Fig. 5A, column 4). When normal LPS-activated splenocytes were co-cultured with adipocytes in transwells to measure the effects of paracrine activity, elevated IL-10 levels were measured; however, this increase was not statistically different from IL-10 secreted by LPS-stimultated splenocytes alone (figure 5A, compare columns 6 and 4). Furthermore, unlike inflammatory cytokines examined above, no statistical difference was found in IL-10 levels generated by co-cultures of splenocytes and adipocytes without contact or with direct cell-cell contact (Fig. 5A, compare columns 6 and 8). These data suggest that both paracrine factors and direct cell-cell contact have little or no influence on IL-10 secretion beyond what is stimulated by LPS treatment.

To further explore if cell-cell contact between splenocytes and adipocytes influences IL-10 expression, we next measured if its mRNA expression was altered following co-culture with direct contact. For this measurement, GFP-expressing splenocytes were once again used for co-culturing with adipocytes to permit FACS separation of the two cell types prior to RNA isolation. By qRT-PCR analyses, we found that direct contact of LPS-activated splenocytes and adipocytes reduced IL-10 splenocyte mRNA expression by 50% (Fig. 5B).

### Signaling through nuclear factor-κB (NF-κB) supports cell contact-mediated enhancement of IL-6 and MCP-1 secretion

**Figure 6:**
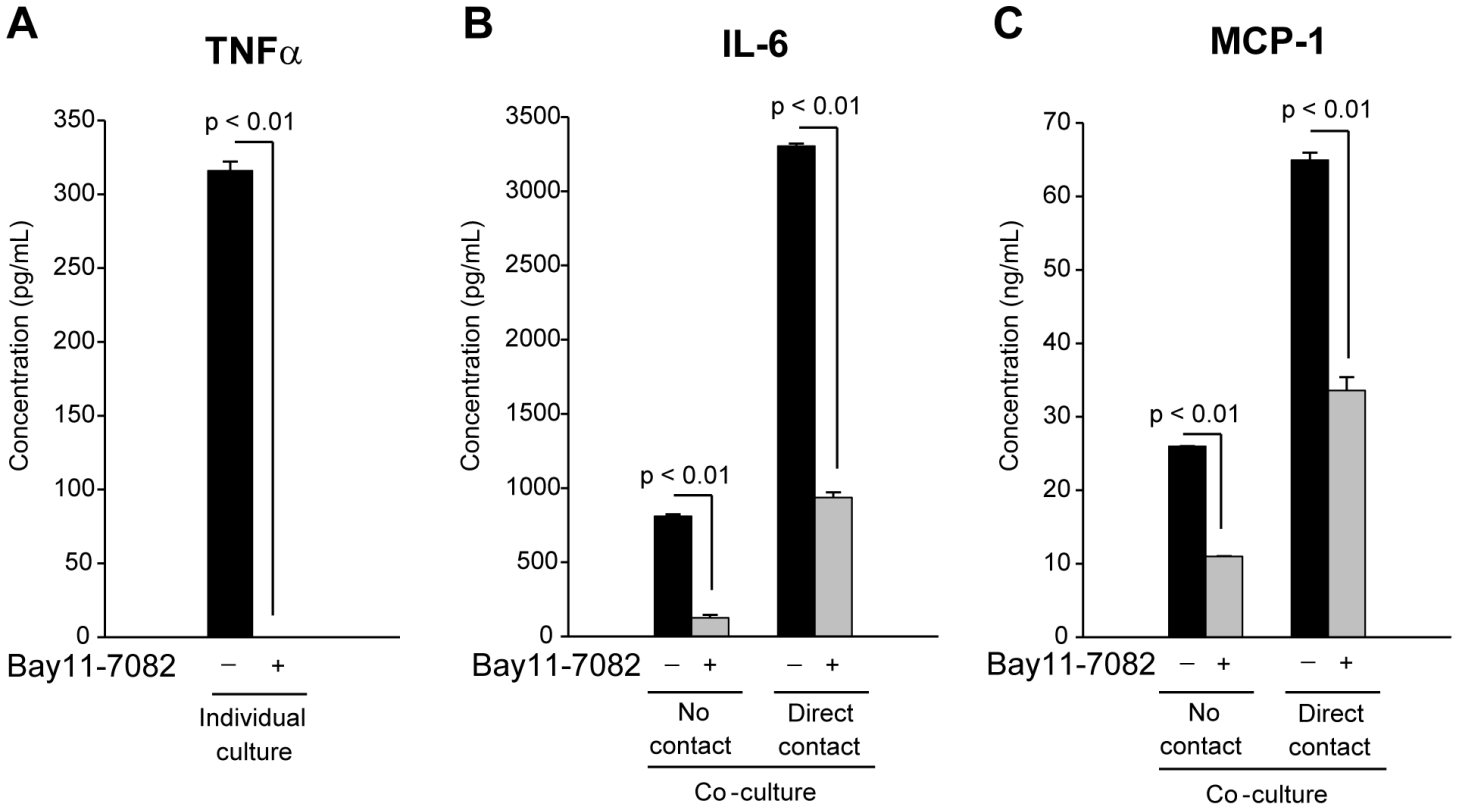
NF-κB intracellular signaling pathway participates in the paracrine and cell contact-mediated enhancement of IL-6 and MCP-1 secretion. (A) Isolated splenocytes were incubated with LPS (1 µg/mL) in the absence (-) or presence (+) of 2 µM Bay11-7082 for 24 h. Secreted levels of TNFα were measured by capture ELISA. (B and C) Differentiated 3T3-L1 adipocytes were co-cultured with isolated splenocytes with either no contact or direct contact as indicated. Cells were stimulated with LPS (1 µg/mL) in the absence (−) or presence (+) of 2 µM Bay11-7082 for 24 h. Secreted levels of IL-6 (B) or MCP-1 (C) were measured by capture ELISA. All experimental points were measured in triplicate for calculation of means and standard deviations. In (A) an unpaired two-tailed Student's t test was used to compare Bay11-7082 treated and untreated LPS-stimulated splenocytes. Comparisons between co-cultures (B and C) were calculated using ANOVA followed by the post-hoc Bonferroni test.

Since we have identified that TNFα activity is necessary for cell contact-mediated enhancement of IL-6 and MCP-1 secretion, we next determined if TNFα provides this function through its normal NF-κB intracellular signaling pathway [45]. For this analysis, we chose to use the well-established chemical inhibitor of the NF-κB pathway, Bay11-7082 [46]. This inhibitor targets the kinase (I-κB kinase, IκK) that releases the cytosolic block on NF-κB and in doing so prevents NF-κB translocation to the nucleus where it performs its transcriptional activation for cytokine gene expression. To confirm the inhibitory activity of Bay11-7082 in our assay, we stimulated splenocytes with LPS in the absence or presence of Bay11-7082 and measured TNFα secretion levels. As anticipated, TNFα secretion was completely inhibited (Fig. 6A) since LPS signaling through TLR4 is known to activate the NF-κB pathway [47].

We next examined if, and to what extent, the NF-κB pathway is involved in the paracrine and cell contact-mediated enhancements we measure for IL-6 and MCP-1 secretion. Treatment of cells with Bay11-7082 inhibited paracrine-mediated enhancement (co-cultures with no cell contact) of IL-6 and MCP-1 secretion by ∼85% and ∼60%, respectively (Fig. 6B and C, no contact). Additionally, the further enhancement of IL-6 and MCP-1 secretion seen when cells are co-culture with direct cell contact was also significantly inhibited by Bay11-7082 to 30% and 50% of control values, for IL-6 and MCP-1 respectively (Fig. 6B and C, direct contact). These findings indicate that the NF-κB pathway is responsible for paracrine stimulation of the majority of IL-6 secretion and a substantial portion of MCP-1. Also, the NF-κB pathway contributes to the cell-contact-mediated enhancement of IL-6 and MCP-1 secretion to a large degree; however, this inhibition is not complete, indicating that other signaling pathways are involved in immune cell-adipocyte crosstalk effects on cytokine secretion.

## Discussion

It is now well established that excessive adiposity in obese individuals is accompanied by a low level, chronic systemic inflammatory state [2], [3]. This observation is clinically important in that chronic secretion of circulating cytokines may be the mechanistic link between obesity and related cardiovascular and diabetic complications; that is, inappropriate cytokine signaling can potentiate atherosclerotic lesion development [48] and, in muscle and adipose tissue, desensitize insulin responsiveness toward glucose clearance function [49]. Elevated circulating cytokines are thought to arise from pro-inflammatory macrophages that populate obese adipose tissue [2], [3]. Recent investigations have also found increased presence of B cells [4], [6], [9], a significant accumulation of CD8^+^ T (effector) cells with a concomitant decrease of CD4^+^ T (helper) cells [4], [5], [7], [8], [10], as well as activated natural killer T (NKT) cells [50], [51]. It has been postulated that B cells provide the initial trigger, leading to activation of CD8^+^ T cells and monocytes/macrophages, which in turn leads to increased inflammation and insulin resistance [9]. To obtain a more detailed understanding of the chronic inflammatory state in obese adipose tissue, we determined if paracrine and cell contact-mediated intercellular communications between immune cells and adipocytes could establish a crosstalk that impacts both cytokine secretion patterns and levels. For this study, we have developed a novel co-culture model using isolated murine splenocytes and cultured murine adipocytes (3T3-L1). The immune cell population in murine splenocytes is known to include CD8^+^ T cells (30%), CD4^+^ T cells (16%), B cells (35%), NKT cells (7%) and a rich source of monocytes (8%) [34], [52]. Most or all of these cells in the spleen have now been identified in obese adipose tissue and likely contribute to some degree toward the pro-inflammatory response. The immune cell distribution in obese adipose tissue has been identified as of CD8^+^ T cells (5%), CD4^+^ T cells (7%), B cells (11%), NKT cells (6%) and macrophages (55%) [35]. Although the percentage representation of each cell type varies between splenocytes and obese adipose tissue, because of the similarity in immune cell identity, use of primary splenocytes in a co-culture model provides a more representative population of immune cells found in obese adipose tissue as opposed to limiting our analyses to a pure population of monocytes/macrophages. 3T3-L1-derived adipocytes have some limitations due to their being an immortalized cell line, as indicated by their storing lipids as multi-locular droplets, as opposed to primary adipocytes, which have uni-locular droplets, as well as secreting very low levels of leptin even when lipid laden. However, we believe these limitations are not significant enough to adversely influence the results of our study, since 3T3-L1-derived adipocytes do correctly respond to endocrine stimulation for fatty acid assimilation and mobilization indicating normal physiologic responses. In addition, these cells demonstrate gene expression profiles that mimic primary adipocyte cultures, especially with regard to metabolic genes needed for proper adipocyte function.

In the present study, we used this novel co-culture model to discriminate between the effects of paracrine signaling from effects mediated by direct cell-cell contact on cytokine secretion in immune cells and adipocytes. We found that when cells are cultured without direct contact, TNFα levels were significantly decreased indicating that soluble factors are secreted in the context of co-cultures that dampen TNFα secretion. Even greater attenuation of TNFα secretion was measured when splenocytes and adipocytes were cultured with direct cell-cell contact. While the maximal effect of paracrine activity reducing TNFα secretion was achieved by 20 h, cell-cell contact provided more rapid signaling to further reduce secretion within 8 h. Paracrine signaling and signaling derived from cell-cell contact appears to act sequentially to decrease TNFα secretion by splenocytes.

In contrast to the effects measured on TNFα secretion, paracrine and direct cell-cell contact have a stimulatory effect on IL-6 and MCP-1 secretion. When splenocytes and adipocytes were co-cultured without direct cell-cell contact, secreted levels of both IL-6 and MCP-1 were significantly increased demonstrating that soluble factors are present that amplify secretion of these cytokines over what can be measured when the two cell types are cultured individually. Even greater increases in IL-6 and MCP-1 secretion were measured when splenocytes and adipocytes were cultured with direct cell-cell contact. While maximal effect of the paracrine activity on IL-6 secretion was achieved by 20 h, cell-cell contact maximally stimulated IL-6 secretion by <8 h. The effects of paracrine stimulation and cell-cell contact on elevating MCP-1 secretion were slower in that the combined effects were unable to reach a maximum even after 48 h of culture. Interpreting these differential effects on IL-6 and MCP-1 is complex. The time course of effects measured for both paracrine and cell-cell contact for IL-6 is similar to those measured for TNFα, although the responses are opposite: IL-6 secretion is increased suggesting transcriptional activation, whereas TNFα is decreased through possible transcriptional repression. Having similar time courses may reflect that the paracrine and cell contact signaling events that determine IL-6 and TNFα secretion changes share overlapping pathways, albeit providing differing effects of either activation or repression, respectively. The effect of paracrine and cell contact signaling on MCP-1 secretion, however, is markedly different suggesting this synergy may require modulation of different regulatory pathways. This interpretation is also compelling when considering the overall level of MCP-1 secretion changes; the quantities of MCP-1 generated in co-cultures are substantially higher than IL-6 indicating a very robust effect on MCP-1 transcriptional/translational activation.

Questions still linger as to which cell type within obese adipose tissue is responsible for the production of each cytokine [53]. Previous attempts have been made to address these questions by disaggregating adipose tissue into adipocytes and stromal vascular cells with proteolysis and culturing cells separately to characterize their cytokine expression profiles. Unfortunately, these attempts have often generated conflicting results, most likely due to disaggregation methods which are now known to alter cytokine expression [33]. Other macrophage-adipocyte co-culture studies have used immortalized macrophage-like cell lines, such as human (THP-1) or murine (RAW264.7) monocytic leukemia cell lines, each having questionable signal transduction pathways because of their transformed phenotype. For these reasons, we performed our study with cells obtained from murine spleens which express normal surface proteins and signaling responses to ensure that our results better represent the cytokine responses we would expect in obese adipose tissue. Following the co-culture of splenocytes obtained from constitutive GFP-expressing mice and adipocytes with direct cell-cell contact, cells were separated by FACS and cytokine expression was determined immediately following sorting to prevent anomalous changes to cytokine profiles. Our data show that the immune cells are the major contributors of TNFα expression, which is consistent with previous findings [26]; however, we also uniquely identified that adipocytes produce most or all of the MCP-1 and approximately one-half of the total IL-6. Together these findings indicate that both cell types make significant contribution towards establishing the chronic inflammatory state in obesity.

Because TNFα is one of the primary macrophage-derived, paracrine-acting cytokines involved in inflammation [26], and considering its potency in increasing expression of other inflammatory mediators such as interleukins, prostaglandins and interferons, it is tempting to speculate that TNFα might have a significant role in establishing, or even enhancing, the chronic inflammation in obese adipose tissue. Some evidence has been reported addressing a role for TNFα in obesity [54] and insulin resistance [55], [56]; however, few studies have examined the effects of TNFα activity on cytokine or adipokine secretion in adipose tissue. We have recently shown that TNFα activates cyclooxygenase-2 expression in adipocytes by activating the NF-κB signaling pathway [27]. This study established that TNFα can activate signal transduction in adipocytes and that this signaling event proceeds through similar pathways as the innate immune response. These findings prompted us to next investigate if endogenously expressed TNFα from splenocytes is able to act in a paracrine manner and mediate the increases in IL-6 and MCP-1 secretion measured in our co-culture system. We also questioned if TNFα can mediate the additional increases we measure on cytokine secretion that result from direct cell-cell contact. By using splenocytes obtained from TNFα deficient (-/-) mice in our co-culture model, we found that when these cells were cultured without direct contact, both IL-6 and MCP-1 secretion was increased to similar levels seen with wild type splenocytes. Additionally, when TNFα ^-/-^ splenocytes are co-cultured with adipocytes in direct cell-cell contact, the additional increases measured for IL-6 and MCP-1 secretion, as seen with direct contact between wild type splenocytes and adipocytes, are absent. Addition of TNFα to these direct co-cultures restored the contact-dependent enhancement of IL-6 and MCP-1 secretion. Together, these data provide evidence that paracrine activity of TNFα contributes little or no function to the initial activation of IL-6 or MCP-1 secretion measured in co-cultures without direct contact, suggesting that soluble factors other than TNFα are largely responsible for driving the paracrine-mediated increases in IL-6 and MCP-1 secretion. While the identities of these alternative soluble factors are currently unknown, they may include novel acute phase reactants produced in adipocytes such as pentaxin-3 [57], [58], which is related to hepatic C-reactive protein. The potential of adipocytes to serve as a potent source of novel acute phase reactants cannot be underestimated. Many of the master gene regulators involved in synthesis of acute phase reactants in liver, such as the members of the C/EBP family, are also abundantly expressed in adipocytes [59].

In contrast to the lack of TNFα activity required for paracrine activation of IL-6 and MCP-1 secretion, we found that TNFα activity is required for cell contact-mediated enhancement of their secretion. The most abundant source of TNFα activity is that which is secreted by immune cells; however, it is also possible that membrane-associated, pro-TNFα is able to engage the TNFα receptor and mimic the effects seen with soluble TNFα [60]. Membrane-associated TNFα is biologically active [61], [62] and provides sufficient activity to induce arthritis in a transgenic model displaying synovial hyperplasia and inflammation [63], [64]. In either case, these findings establish a novel role for TNFα in adipose tissue inflammation and define a cellular mechanism whereby TNFα fuels the inflammatory response activated by immune cell-adipocyte contact.

The intracellular signaling events of this unique TNFα activity likely involve crosstalk between signaling pathways in adipocytes. Co-culture of splenocytes and adipocytes can engage cell surface molecules which could in turn activate intracellular signaling pathways that converge with TNFα receptor signaling. By this convergence, TNFα signaling may sustain or amplify signals initiated from surface receptor engagement and support elevated cytokine secretion levels. The link between these cell surface events is likely to involve communication between known pathways; for example, use of specific inhibitors to block mitogen-activated protein (MAP) kinase pathways (primarily ERK and JNK) and NF-κB activation was able to prevent the overall inflammatory response seen in co-culture studies of adipocytes and macrophages [26], [65], or human adipose tissue [66]. However, these data were limited in that they only demonstrated global effects on cytokine production and did not address if inhibition of these inflammatory changes was due to inhibition of pathways in adipocytes, macrophages, or both. Other studies showed that stimulation of adipocytes with TNFα or cell-cell contact between adipocytes and immortalized macrophages can lead to up-regulation of the NF-κB pathway [65], [67]. By using a well established chemical inhibitor of the NF-κB pathway, we have identified that this pathway substantially contributes to the measurable enhancement of IL-6 and MCP-1 secretion that occurs through both paracrine stimulation and cell-cell contact. However, inhibition of NF-κB is not sufficient to prevent all secretion indicating that other pathways significantly participate in immune cell-adipocyte crosstalk leading to an enhanced inflammatory response. It is likely that TNFα receptor engagement could activate all or a combination of MAP kinase, NF-κB, Jak2 and p44/42 pathways to communicate in a synergistic manner with the NF-κB pathway to enhance a pro-inflammatory state in obese adipose tissue. The data we present here shows that paracrine factors and direct cell-cell contact are at the center of coordinating the activities of these signaling pathways to direct the pattern and intensity of the inflammatory process.

Since increases in IL-10 secretion are responsive to the elevated state of inflammation within obese adipose tissue [42], [43], we also examined the effects of paracrine stimulation and direct contact on IL-10 secretion in our co-culture system. We found that co-culture of splenocytes and adipocytes without direct contact yielded IL-10 secretion levels that were not statistically different from individual cultures of LPS-stimulated splenocytes and adipocytes; suggesting that paracrine factors have little or no influence on IL-10 levels, rather LPS activation is sufficient to induce IL-10 secretion. Moreover, unlike inflammatory cytokines examined above, no change in IL-10 levels was found when splenocytes and adipocytes were co-cultured with direct contact. Interesting, direct contact of splenocytes with adipocytes was able to reduce IL-10 mRNA expression by 50% of what was measured for co-cultures without direct contact, indicating that cell contact does either dampen IL-10 transcription or reduce mRNA half-life. Clearly these data show that cell-cell contact affects IL-10 mRNA levels, but this effect is not reflected in secreted protein levels.

It is clear that immune cell infiltration into obese adipose tissue is fundamental for changes measured in cytokine secretions and that there is crosstalk between these cells and resident adipocytes. Our findings here allow us to now postulate that there are specific, complementary cell surface molecules expressed on both cell types, that when engaged, can cause significant modifications to cytokine secretion profiles, in conjunction with the diffusible factors that are already being secreted into the local environment. From this novel identification, we anticipate that a more complete understanding of the cell surface and intracellular signaling events that mediate this effect might provide a novel therapeutic dimension targeted to reduce inflammation in obese adipose tissue.
